# Vascular smooth muscle cells direct extracellular dysregulation in aortic stiffening of hypertensive rats

**DOI:** 10.1111/acel.12748

**Published:** 2018-03-30

**Authors:** Tristan T. Hays, Ben Ma, Ning Zhou, Shaunrick Stoll, William J. Pearce, Hongyu Qiu

**Affiliations:** ^1^ Division of Physiology Department of Basic Sciences School of Medicine Loma Linda University Loma Linda CA USA

**Keywords:** aortic stiffness, extracellular matrix, hypertension, lysyl oxidase, vascular smooth muscle cells

## Abstract

Aortic stiffening is an independent risk factor that underlies cardiovascular morbidity in the elderly. We have previously shown that intrinsic mechanical properties of vascular smooth muscle cells (VSMCs) play a key role in aortic stiffening in both aging and hypertension. Here, we test the hypothesis that VSMCs also contribute to aortic stiffening through their extracellular effects. Aortic stiffening was confirmed in spontaneously hypertensive rats (SHRs) vs. Wistar‐Kyoto (WKY) rats *in vivo* by echocardiography and *ex vivo* by isometric force measurements in isolated de‐endothelized aortic vessel segments. Vascular smooth muscle cells were isolated from thoracic aorta and embedded in a collagen I matrix in an *in vitro* 3D model to form reconstituted vessels. Reconstituted vessel segments made with SHR VSMCs were significantly stiffer than vessels made with WKY VSMCs. SHR VSMCs in the reconstituted vessels exhibited different morphologies and diminished adaptability to stretch compared to WKY VSMCs, implying dual effects on both static and dynamic stiffness. SHR VSMCs increased the synthesis of collagen and induced collagen fibril disorganization in reconstituted vessels. Mechanistically, compared to WKY VSMCs, SHR VSMCs exhibited an increase in the levels of active integrin β1‐ and bone morphogenetic protein 1 (BMP1)‐mediated proteolytic cleavage of lysyl oxidase (LOX). These VSMC‐induced alterations in the SHR were attenuated by an inhibitor of serum response factor (SRF)/myocardin. Therefore, SHR VSMCs exhibit extracellular dysregulation through modulating integrin β1 and BMP1/LOX via SRF/myocardin signaling in aortic stiffening.

## 1 INTRODUCTION

Aortic stiffening is a fundamental component of aging‐related vascular diseases (McEniery, Wilkinson & Avolio, 2007; Wallace et al., 2007). Increased aortic stiffness, whatever the underlying cause, is also an independent predictor of outcomes of cardiovascular diseases in the elderly. It is well known that hypertension is a highly age‐related human disease. Despite a widely held belief that increased aortic stiffness in hypertensive patients is largely a manifestation of long‐standing hypertension‐related damage, a recent statement from the American Heart Association (AHA) asserts that aortic stiffening is a cause rather than a consequence of hypertension in middle‐aged and older individuals (Townsend et al., 2015). This new concept further clarifies the cause and effect relationship between aortic stiffening and hypertension in aged individuals. Additionally, our previous studies in monkey have demonstrated that aortic stiffening is also strongly associated with aging (Qiu et al., 2007). Specifically, our recent studies with atomic force microscopy (AFM) have demonstrated similar characteristics of aortic vascular smooth muscle cells (VSMCs) in both aging and hypertension, indicating that VSMC‐mediated regulation is a fundamental basis of aortic stiffening in both conditions (Qiu et al., 2010; Zhou, Lee, Stoll, Ma, Costa, et al. 2017; Zhou, Lee, Stoll, Ma, Wiener, et al. 2017). However, the underlying mechanisms are not fully understood. It is conceivable that, in addition to intracellular effects, VSMCs are able to contribute to aortic stiffening via extracellular effects. However, it is difficult to discern the extracellular effects of VSMCs in intact aortic tissue *in vivo*. Our previous study successfully distinguished the role of VSMCs from the extracellular matrix (ECM) in aortic stiffness *in vitro* utilizing a three‐dimensional (3D) tissue model reconstituted system consisting of isolated VSMCs and collagen (Qiu et al., 2010). This model also provides the necessary simplicity to characterize interactions between VSMCs and the surrounding ECM and explore the molecular mechanisms mediating these changes.

In our previous study, integrin β1 was found to be significantly increased in VSMCs from stiffened aortas in aging monkeys (Qiu et al., 2010), indicating that integrin β1 may contribute to aortic stiffening. Other recent studies emphasize the potential role of Lysyl oxidase (LOX), a copper‐dependent amine oxidase, in vascular remodeling and the regulation of the biomechanical properties of the ECM (Rodriguez et al., 2008). The LOX gene encodes a precursor protein (pre‐LOX), which is proteolytically processed by bone morphogenetic protein‐1 (BMP1) and other proteinases to release the mature active enzyme LOX (M‐LOX) and the LOX regulatory pro‐peptide (LOX‐PP) (Rodriguez et al., 2008). The extracellular active enzyme catalyzes formation of aldehydes from lysine residues in collagen and elastin precursors resulting in cross‐linking among these ECM proteins that stabilize collagen fibrils and maintain the integrity and elasticity of mature elastin. Different patterns of LOX expression/activity have been associated with distinct vascular pathological processes. For example, downregulation of LOX has been associated with destructive remodeling of arteries during aorta aneurysm (AA) development (Yoshimura et al., 2006). Deletion of the mouse LOX gene promotes fragmentation of elastic fibers and VSMC discontinuity in the aortic wall, leading to increased impedance and a predisposition to thoracic AAs and dissections (Maki et al., 2002; Staiculescu, Kim, Mecham & Wagenseil, 2017). Loss‐of‐function mutations of LOX can cause AAs and aortic stiffening in humans (Lee et al., 2016). These studies indicate an essential role of LOX in maintaining the tensile and elastic features of blood vessels. Studies of the role of LOX in hypertensive aortic stiffening, however, have produced inconsistent results (Chen et al., 2013; Eberson et al., 2015).

The present study tests our hypothesis that aortic VSMCs contribute to aortic wall stiffness via both increased intrinsic stiffness and extracellular dysregulation mediated through altered regulation of integrin and LOX signaling. Given that our recent study revealed a pivotal role for upregulation of the serum response factor (SRF)/myocardin pathway in pathological aortic stiffening (Zhou, Lee, Stoll, Ma, Costa, et al. 2017; Zhou, Lee, Stoll, Ma, Wiener, et al. 2017), we further explored possible links between SRF/myocardin and integrin/LOX signaling in the extracellular regulation of stiffness by aortic VSMCs.

## 2 RESULTS

### 2.1 VSMCs contribute to aortic stiffening in SHR

To determine the role of VSMCs in hypertensive aortic stiffening, we conducted a series of experiments in both spontaneously hypertensive rats (SHRs) and their normotensive controls, Wistar‐Kyoto (WKY) rats, to measure aortic stiffness *in vivo*, in de‐endothelialized native aortic rings *ex vivo*, and in 3D reconstituted rings *in vitro*.

**Figure 1:**
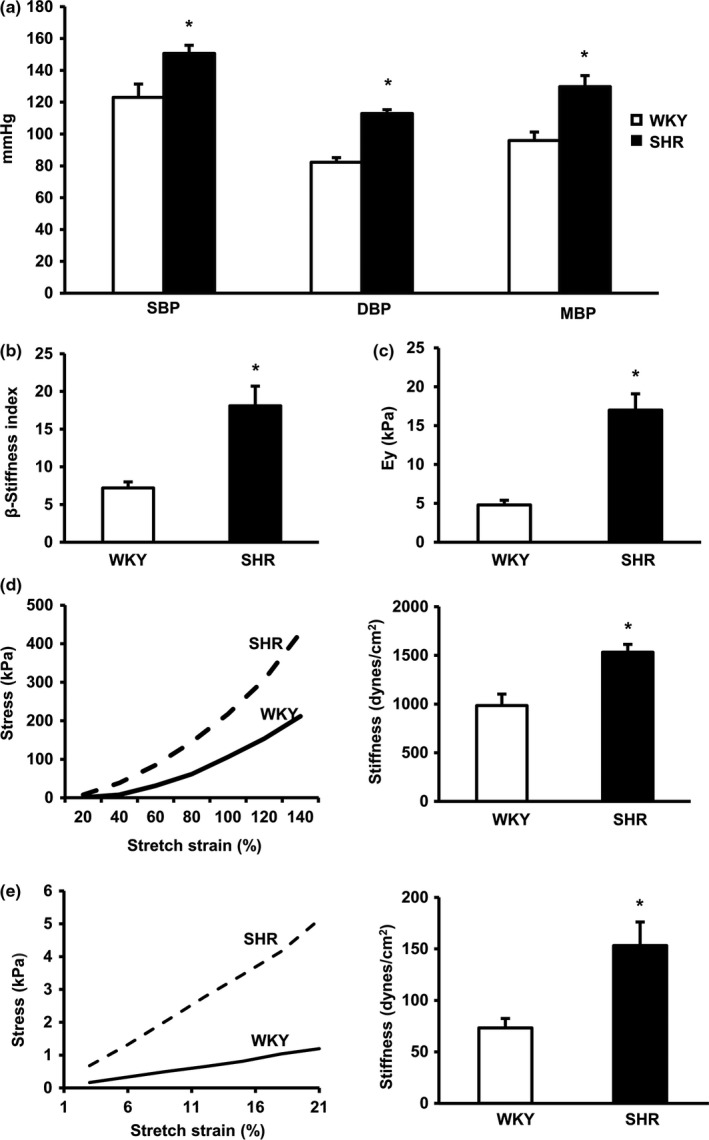
Aortic stiffness is increased in spontaneously hypertensive rats (SHR) *in vivo*,* ex vivo*, and *in vitro* 3D reconstituted tissue. (a) Systolic and diastolic blood pressure (SBP and DBP) as well as the calculated mean blood pressure (MBP) in spontaneously hypertensive rats (SHRs) and their age‐matched normotensive controls, Wistar‐Kyoto (WKY) rats. *n* = 15 rats/group. (b, c) Aortic stiffness measured *in vivo* by echography, represented by stiffness index (β) (b) and Young's modulus (EY) (c) *n* = 15 rats/group. (d, e) Representative stress–strain response curve and calculated stiffness in the medial layer of isolated native vessel segments measured *ex vivo* (d) and in 3D reconstituted tissue rings measured by an isometric force transducer (e) * *p *<* *.01 vs. WKY. *n* = 15 rings/group (3 rings/rat for five rats/group)

Given that increased stiffness in SHR aortas is the physiological foundation of the *ex vivo* and *in vitro* studies, we first performed *in vivo* measurements to confirm aortic stiffing in these SHRs. Both systolic blood pressure and diastolic blood pressure (SBP and DBP), as well as mean blood pressure (MBP), were increased in adult SHRs compared to age‐matched normotensive WKY rats when measured via tail cuff under conscious conditions (Figure 1a) and confirmed by a Millar catheter under anesthesia. Aortic stiffness, measured with Doppler echocardiography *in vivo*, was significantly increased in SHR vs. WKY rats, as indicated by a 2.5‐fold increase in the stiffness index (β) (Figure 1b) and a 3.5‐fold increase in Young's modulus (E_Y_) (Figure 1c).

To determine if increased aortic stiffness in SHRs was independent of any neural‐hormone effects or from the endothelial layer *in vivo*, aortic tissue segments were isolated from the same animals, cleaned of extraneous connective tissue, denuded of the endothelia, and used to measure aortic stiffness *ex vivo* with an isometric force transducer. Consistent with increased aortic stiffness *in vivo*, fresh de‐endothelialized aortic rings were also significantly stiffer in SHR vs. WKY rats (Figure 1d), indicating a stiffer medial layer in SHR aortas.

To further define the contributions of VSMCs to aortic stiffness independent of the external influence of ECM proteins, a 3D reconstituted vessel model with VSMCs from the thoracic aorta (TA) was used in which the artificial ECM was identical in both groups. The resulting artificial vessel segments were subjected to uniaxial tensile stretching and the construct's stiffness (E) was calculated as described above in the native aortic ring. As shown in Figure 1e, reconstituted rings were twofold stiffer when reconstituted with VSMCs from SHR than those from WKY rats.

Together, these data indicate that aortic VSMCs contribute significantly to aortic stiffening in SHRs independent of endothelial cells and ECM in the medial layer.

### 2.2 SHR VSMCs influence both static and dynamic stiffness in 3D reconstituted rings

In our previous study, AFM revealed that compared to WKY TA VSMCs, SHR TA VSMCs increased intrinsic stiffness at the single cell level by 1.5 fold (Zhou, Lee, Stoll, Ma, Wiener, et al. 2017). Interestingly, even greater differences (more than twofold) in stiffness were observed when VSMCs from SHR TA and WKY TA were embedded in 3D reconstituted tissue constructs. The greater increase in stiffness of the reconstituted tissue over that of the single cells would imply additional outward effects of the cells upon interactions with the ECM. Thus, we next tested the extracellular effects of VSMCs.

**Figure 2:**
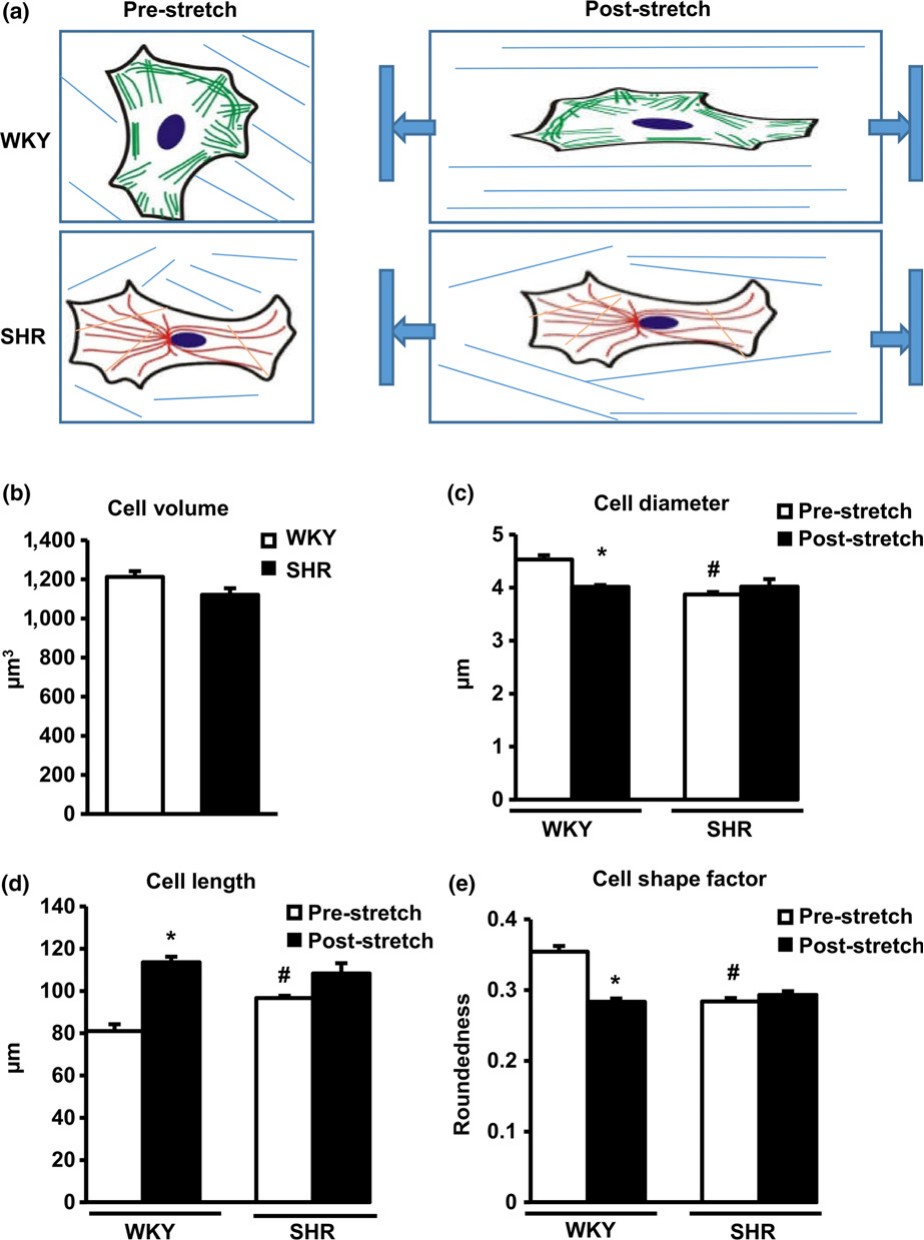
Vascular smooth muscle cells (VSMCs) mediate extracellular interactions in 3D reconstituted rings. (a) The representative illustration of VSMC morphology in reconstituted rings. (b) Cell volumes in 3D reconstituted rings. * *p *<* *.01 vs. Wistar‐Kyoto (WKY). *n* = 12 rings/group (3 rings/rat for four rats/group). (c–e) The indexes of cell shape of VCMCs in 3D reconstituted rings and the adaptability under the stress. * *p *<* *.01 vs. prestretched ring, ^#^ *p *<* *.01, vs. corresponding WKY. *n* = 12 rings/group (3 rings/rat for four rats/group)

To determine the interactions between cells and the ECM in SHR TA, we conducted microscopic studies in 3D reconstituted rings to determine changes in VSMCs when they were in contact with ECM under baseline and stressed conditions. Isolated TA VSMCs from both SHR and WKY rats were seeded in a collagen I gel in a 3D mold to generate artificial vessel segments. After 48 hr of culture, the resulting artificial segments were subjected to stepwise uniaxial tensile stretching up to 120% of their original resting length for 1‐min periods. Embedded VSMCs were then stained for αSMA and imaged by two‐photon confocal microscopy. Z‐stack images were acquired to construct a 3D model. Vascular smooth muscle cell cellular dimensions were measured. As illustrated in Figure 2a, cell shape differed significantly between VSMCs for SHR TA and WKY TA after stretching despite similar cell volumes (Figure 2b). Embedded SHR TA VSMCs exhibited smaller diameters (Figure 2c) appeared more elongated (Figure 2d) and were less rounded (Figure 2e) in 3D reconstituted tissue compared to WKY TA VSMCs. After stretching the reconstituted tissue rings, the embedded WKY VSMCs adapted to load and became thinner and longer when stretched, whereas SHR TA VSMCs were more resistant to morphological changes induced by force (Figure 2a–e). This resistance to deformation further confirms that the increased stiffness of VSMCs from SHR TA vs. WKT TA previously found via AFM is also true in 3D in a more physiologically representative state.

### 2.3 VSMCs induce collagen synthesis and fibril disorganization in the SHR TA

**Figure 3:**
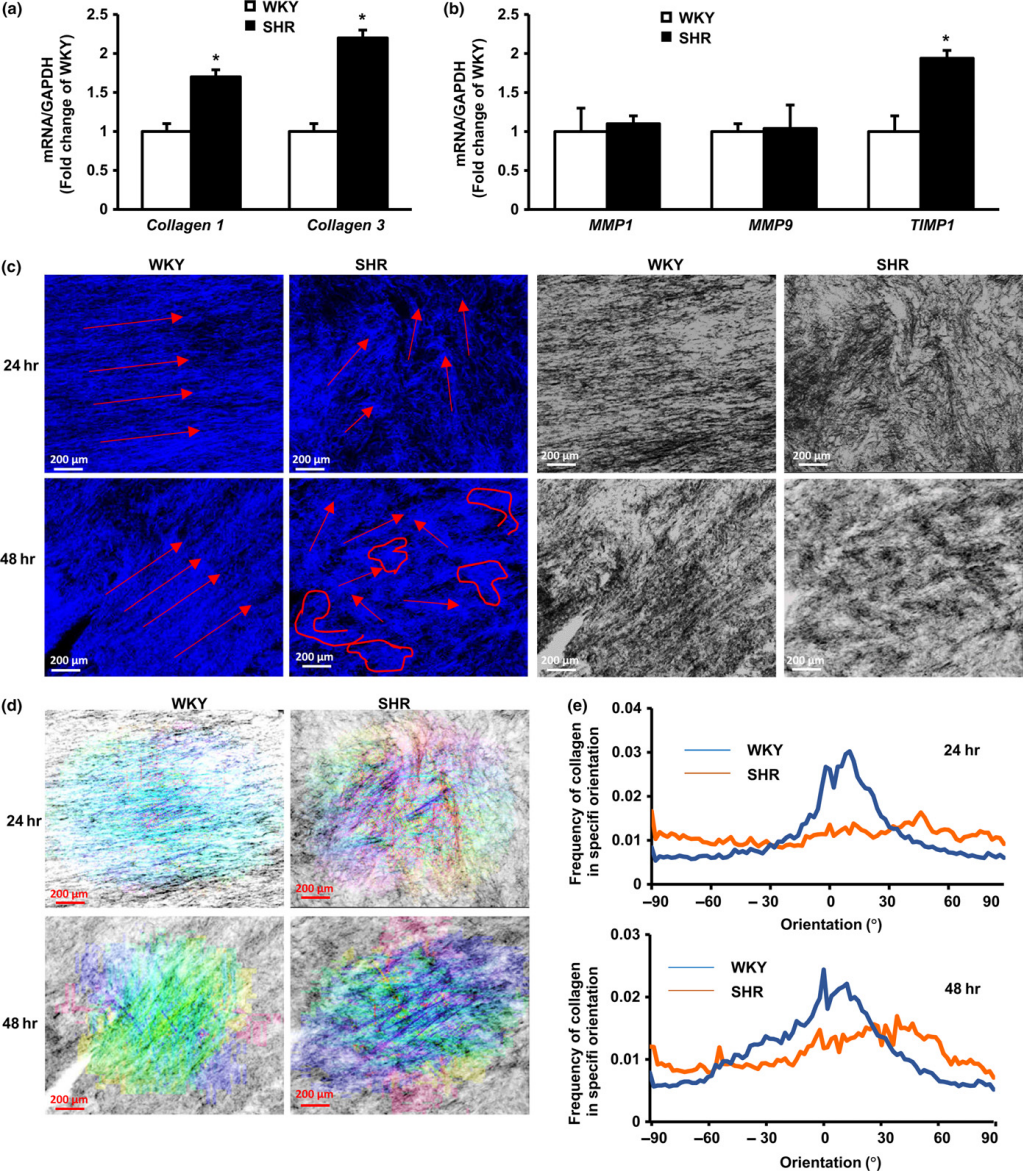
Vascular smooth muscle cells (VSMCs) induce collagen synthesis and disorganization in spontaneously hypertensive rats (SHR) aorta. (a, b) The ratio of mRNA levels of collagen types I and III, and MMPs/TIMP with GAPDH in VSMCs from SHR vs. Wistar‐Kyoto (WKY) rats. * *p *<* *.01 vs. WKY. *n* = 4/group rats with a triplication of each sample. (c) The representative images of collagen in 3D reconstituted rings by second harmonic generation (SHG) microscopy, showing the organization and orientation of collagen fibers. (d) Fourier component analysis for directionality image analysis showing the orientation of collagen in 3D reconstituted tissues. (e) The quantitative frequency of collagen directionality

To determine the effects of VSMCs upon the ECM, we probed the production of the two major isoforms of aortic collagen by measuring the synthesis of collagens I and III. As shown in Figure 3a, mRNA levels for *collagens I* and *III* were significantly higher in VSMCs from SHR TA compared to WKY TA. When we determined the potential effects of VSMCs upon collagen degradation by measuring matrix metalloproteinases (MMPs), and their inhibitors (TIMPs), there was a significant increase in *TIMP1* mRNA in VSMCs from SHR TA compared to WKY TA, but no differences in mRNA levels of *MMP1* and *MMP9* (Figure 3b).

Next, VSMCs were cultured with collagen I in a 3D model for 24 or 48 hr to generate reconstituted vessel rings. Collagen in these rings was imaged using 2‐photon second harmonic generation (SHG) microscopy. After 24 hr, collagen cultured with WKY TA VSMCs exhibited a parallel alignment, whereas collagen cultured with SHR TA VSMCs showed significant disarray with greater alignment angles. This collagen disorganization included shorter collagen fragments and was more pronounced after 48 hr with SHR TA VSMCs. In contrast, 48 hr of culture with WKY TA VSMCs produced no significant changes in collagen organization (Figure 3c). The assembly, organization, and directionality of collagen fibrils were further determined by Fourier component analysis (Figure 3d). Collagen fibrils in reconstituted tissue embedded with WKY TA VSMCs were primarily aligned in one dominant direction, whereas fibrils in cultures with SHR TA VSMCS were oriented in multiple directions after 24 hr. These differences in collagen organization between SHR and WKY became more pronounced after 48 hr (Figure 3e). Together, these data indicate that SHR TA VSMCs influence collagen synthesis and degradation and also disrupt collagen organization.

### 2.4 VSMCs regulate integrin β1 in SHR TA

**Figure 4:**
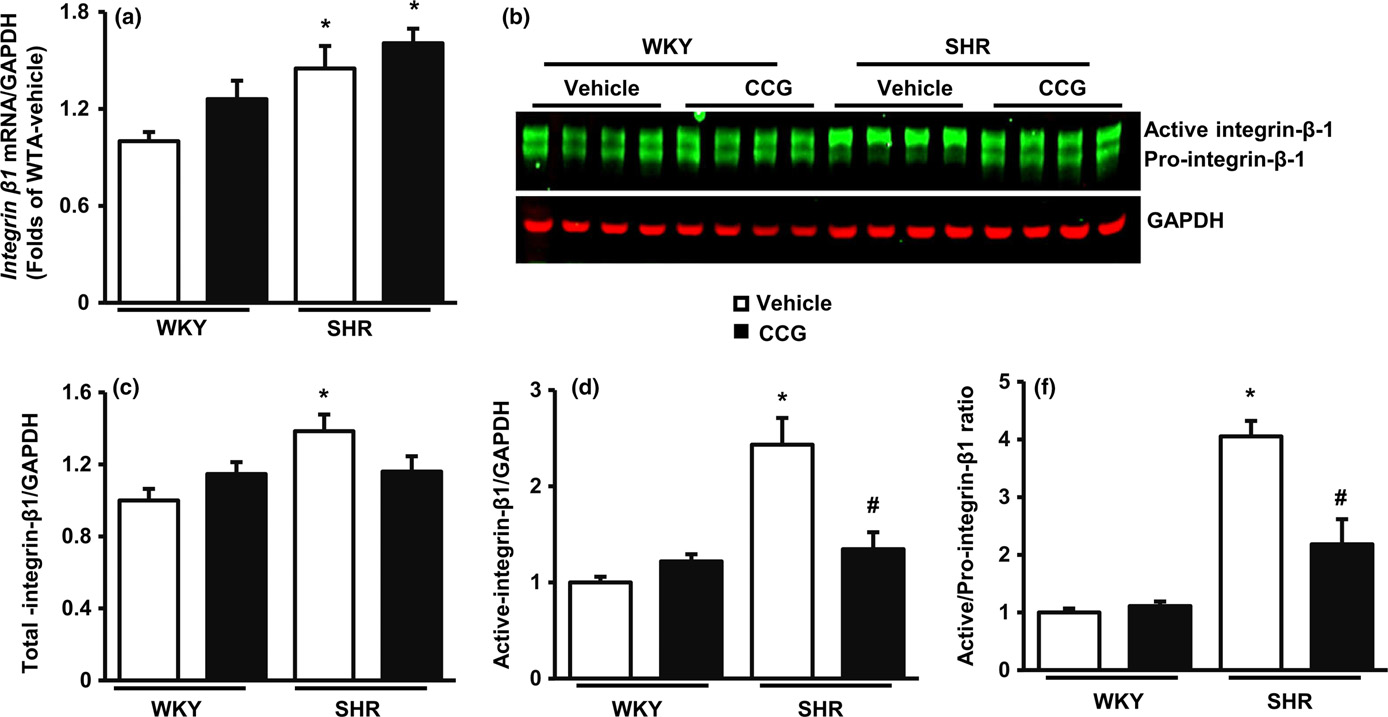
Vascular smooth muscle cells (VSMCs) increase the expression and activation of integrin β1 in spontaneously hypertensive rats (SHR) aorta. (a) The relative RNA level of integrin β1 in TA VSMCs from SHR vs. Wistar‐Kyoto (WKY) rats. *N* = 8/group. (b) Examples of Western blots of integrin β1 subunits. GAPDH was used as a loading control. (c–e) The average value of integrin β1 expression in terms of total protein (c), active forms (d), and the ratio of active to pro‐ integrin β1 forms (e). * *p *<* *.01 vs. WKY. ^#^ *p *<* *.01 vs. corresponsive vehicle treatment. *N* = 8/group

To explore the molecular mechanisms underlying VSMC‐mediated cell‐ECM interactions, we measured the expression of integrin β1 subunits in VSMCs. The mRNA level of *integrin* β*1* measured by qPCR was moderately higher in VSMCs from SHR TA compared to WKY TA (Figure 4a). Consistent with other reports (She, Xu, He, Lan & Wang, 2010), two forms of integrin β1 protein were detected via Western blot, including both precursor (lower band) and active forms (upper band) (Figure 4b). Compared to VSMCs from WKY TA, those from SHR TA showed a 1.4‐fold increase in total integrin β1 (Figure 4c), a 2.4‐fold increase in active integrin β1 (Figure 4d), and a significant increase in the ratio of active to precursor integrin isoforms (Figure 4e).

Treatment with the SRF/myocardin inhibitor CCG‐100602 (CCG) as previously described (Zhou, Lee, Stoll, Ma, Costa, et al. 2017; Zhou, Lee, Stoll, Ma, Wiener, et al. 2017) had no effect on integrin β1 mRNA relative to vehicle controls in cells from either WKY or SHR aorta (Figure 4a). The abundance of activated integrin β1 protein was markedly attenuated by CCG treatment in VSMCs from SHR TA, but not those from WKY TA (Figure 4b–e).

### 2.5 VSMCs regulate LOX in SHR TA

**Figure 5:**
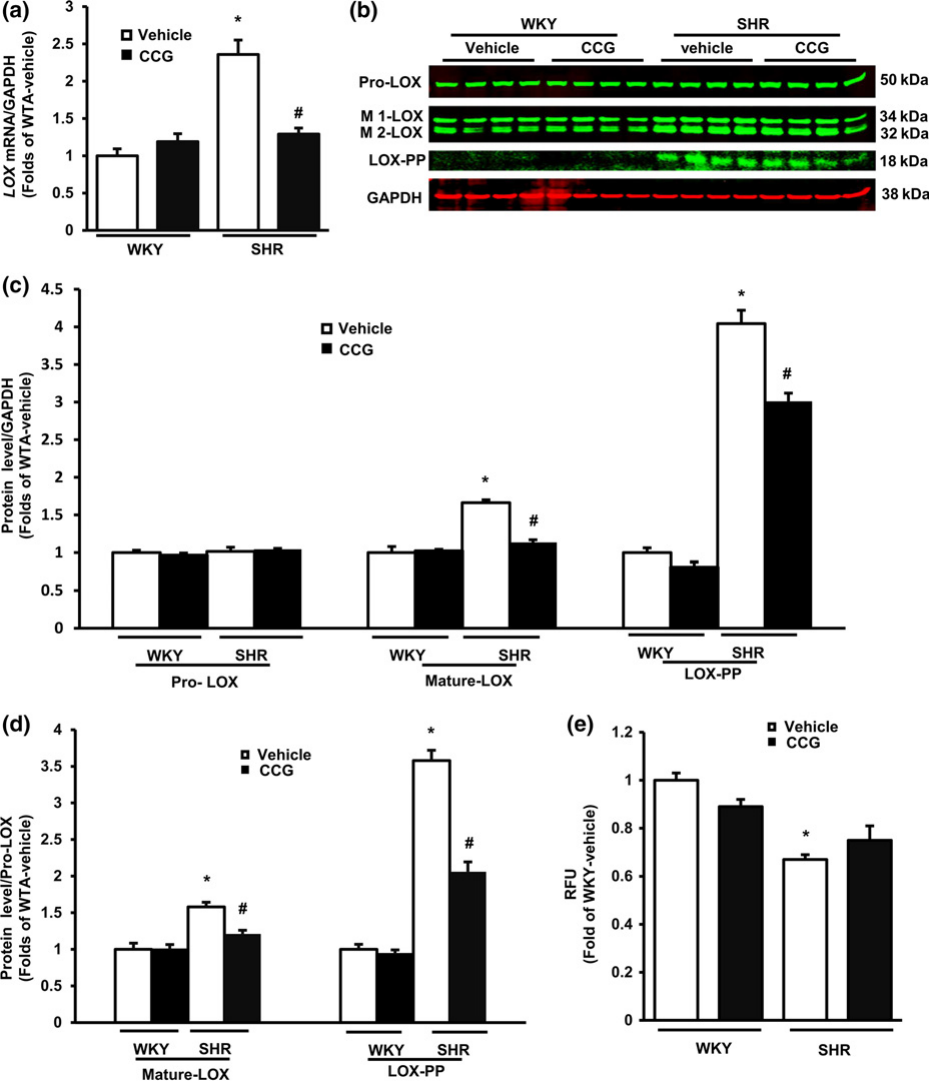
Vascular smooth muscle cells (VSMCs) regulate Lysyl oxidase (LOX) production in spontaneously hypertensive rats (SHR) aorta. (a) The mRNA level of LOX in VSMCs. *N* = 8/group. (b) Examples of Western blots of LOX isoforms in VSMCs. GAPDH: a loading control. (c) Relative protein levels of LOX/GAPDH and (d) the ratio of mature‐LOX and LOX‐PP of Pro‐LOX. *N* = 8/group. (e) Activity of LOX in VSMC cultured medium. *N* = 8/group. * *p *<* *.05 vs. Wistar‐Kyoto. ^#^ *p *<* *.05 vs. vehicle control

Lysyl oxidase mRNA levels were found to be significantly higher in VSMCs from the SHR TA than from WKY TA (Figure 5a). Western blotting detected four LOX isoforms, including pro‐LOX (~50 kDa), two mature‐LOX isoforms (~34 kDa and ~32 kDa), and LOX pro‐peptide (LOX‐PP, ~18 kDa) (Figure 5b). The abundance of pro‐LOX did not differ significantly between SHR and WKY TA VSMCs. However, the mature‐LOX and LOX‐PP forms were significantly more abundant in VSMCs from SHR TA than from WKY TA (Figure 5c). The ratios of mature‐LOX/pro‐LOX and LOX‐PP/pro‐LOX were also significantly higher in VSMCs from SHR TAs than from WKY TAs (Figure 5d). In aggregate, these findings suggest increased rates of proteolytic cleavage of LOX in VSMCs from SHR compared to WKY animals.

CCG treatment decreased *LOX* mRNA expression in VSMCs from SHR TA and not from WKY TA (Figure 5a). Despite no effect on pro‐LOX protein levels in either group (Figure 5c), CCG treatment significantly reduced the levels of mature‐LOX and LOX‐PP in SHR VSMCs vs. vehicle control (Figure 5c), and thus reduced the ratios of mature‐LOX and LOX‐PP to pro‐LOX in SHR TA VSMCs (Figure 5d). These effects of CCG, however, were absent in VSMCs from WKY TA (Figure 5c–d). Additionally, the activity of LOX secreted into the culture media was significantly less for VSMCs from SHR compared to WKY cultures (Figure 5e). CCG treatment had no significant effects upon extracellular LOX activity in either group.

### 2.6 VSMCs regulate LOX via BMP1

**Figure 6:**
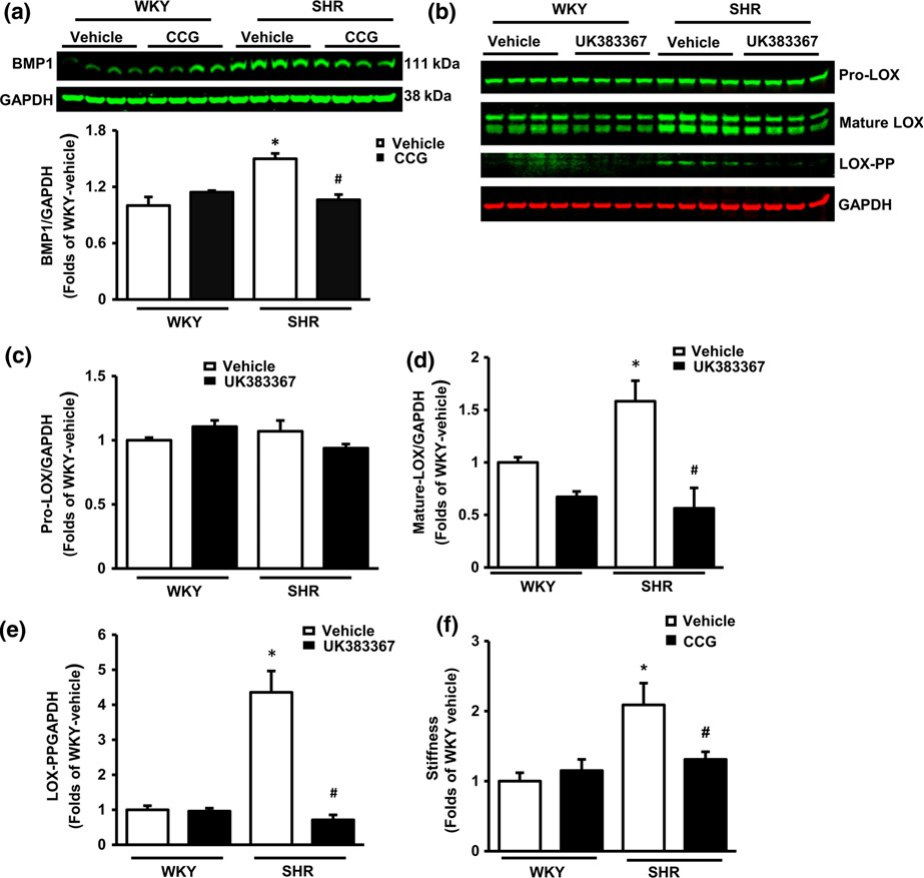
Vascular smooth muscle cells (VSMCs) regulate BMP1 in the spontaneously hypertensive rats aorta. (a) Western blotting and relative protein levels of BMP1 in VSMCs. (b) The representative Western blotting of Lysyl oxidase (LOX) in VSMCs. (c‐e) Relative protein levels of LOX isoforms in VSMCs. *N* = 4/group. * *p *<* *.01 vs. Wistar‐Kyoto (WKY). ^#^ *p *<* *.01 vs. vehicle control. GAPDH: a loading control. (f) The relative level of stiffness of reconstituted rings upon treatment with CCG or vehicles measured by an isometric force transducer. *n* = 10 rings/group (2 rings/rat for five rats/group). *p <* *.01 vs. WKY. ^#^ *p *<* *.01 vs. vehicle control

We further explored how SRF/myocardin signaling might regulate LOX proteolytic cleavage in VSMCs. As shown in Figure 6a, levels of BMP1, a protease that targets LOX, were significantly greater in VSMCs from SHR than from WKY. Treatment with CCG, which inhibits SRF/Myocardin, attenuated the increased levels of BMP1 in VSMCs from SHR, suggesting a link between upregulation of SRF/myocardin and the increased activity of BMP1. Vascular smooth muscle cells from both SHR and WKY aorta were then treated with a selective inhibitor of BMP1, UK383367. As shown in Figure 6, UK383367 did not alter Pro‐LOX levels in either group, but significantly attenuated levels of mature‐LOX (Figure 6d) and LOX‐PP (Figure 6e) in SHR VSMCs, but not in cells from WKY; similar to the pattern produced by CCG treatment (Figure 5c–d). Finally, we tested if inhibition of SRF/myocardin could reduce the stiffness of reconstituted tissue *in vitro*. CCG treatment (25 μm) for 24 hr was found to significantly reduce the stiffness of the reconstituted tissue formed with VSMCs from SHR TA, but not from WKY TA (Figure 6f).

## 3 DISCUSSION

This study integrated *in vivo*,* ex vivo*, and *in vitro* experiments to identify the individual components that contribute to aortic stiffness. We first determined the contributions of the crucial medial layer to aortic stiffening, which is independent of endothelium and adventitia. Second, use of a 3D reconstituted vessel revealed that VSMCs are essential for aortic stiffening, independent of the ECM in the medial layer. The data from both *ex vivo* native tissues and *in vitro* reconstituted tissues complemented our previous studies of single VSMCs (Zhou, Lee, Stoll, Ma, Wiener, et al. 2017) and emphasize the importance of VSMCs in aortic stiffening.

Since the initial cell number and amount of collagen are the same for SHR and WKY rat groups in our 3D reconstituted tissue model, the VSMCs' function contributes to the difference between two groups that occurred during the ring formation, including the impacts on the interaction of VSMCs with ECM and on the synthesis and remodeling of ECM. Our data demonstrated that, when surrounded by the collagen, SHR TA VSMCs exhibited different morphologies at baseline condition and diminished the adaptability to stress compared to WKY VSMCs. These data imply the dual roles of VSMCs in both static and dynamic stiffness at a tissue level. These characteristics of TA VSMCs in hypertension have not been observed *in vivo* previously; thus, our results provide new evidence showing that the phenotypic transformation of VSMCs is a key component of SHR TA that contributes to aortic stiffening in hypertension.

In addition to the differences in interacting with the ECM between SHR VSMCs and WKY VSMCs, we further demonstrated that aortic VSMCs from SHR are able to affect the ECM through the regulation of collagen production and the organization of the ECM. Compared to WKY, SHR TA VSMCs showed a higher level of mRNA level of collagen types I and III, the two predominant isoforms of collagen in the aorta, which indicates an increase in collagen synthesis in VSMCs from stiffer aortas. The TIMP1/MMP ratio was also significantly increased in SHR TA VSMCs vs. WKY TA VSMCs, which were consistent with the previous observation of aortic tissues in SHRs (Duan et al., 2015). Since TIMP1 is a well‐recognized inhibitor of MMPs, which participate in collagen degradation (Duan et al., 2015), this increase in TIMP‐1 induced by SHR aortic VSMCs might contribute to the unfavorable collagen accumulation in the aortic wall (Zhou, Lee, Stoll, Ma, Wiener, et al. 2017). In addition, using the 3D reconstituted model, we revealed that SHR TA VSMCs induced collagen disarray in both orientation and fragmentation. These data provide direct evidence that SHR TA VSMCs are able to impact collagen construction in aortic tissue. These results from the *in vitro* tissue resemble ECM topography detected in stiffening aorta and other arteries *in vivo* (Dodson et al., 2013; Harvey, Montezano, Lopes, Rios & Touyz, 2016) and thus emphasize the effect of VSMCs upon ECM remodeling and contribute to the aortic stiffening in SHR.

Therefore, our data together demonstrated that the contribution of SHR VSMCs to aortic stiffness has two components: intrinsic and extrinsic. The intrinsic component results from the alteration of the VSMC itself, including increased stiffness of the individual cell and decreased adaptability to the ECM. The extrinsic component is the modulation of ECM by VSMCs, via both synthesis and degradation of collagens, and by facilitating the reorganization of ECM.

Furthermore, we investigated the potential molecular basis behind VSMC‐mediated extracellular effects in the stiffening aorta of SHRs. Two major alterations were found in our mechanistic study: the upregulation in activation of integrin β1 and in LOX maturation in SHR VSMCs vs. WKY VSMCs. First, our results showed an increase in integrin β1 induced in SHR TA VSMCs at the both mRNA and protein levels. This result is consistent with our previous observation in the stiffer aorta in aging animals (Qiu et al., 2010). In addition, we found that activated integrin β1 was significantly increased in SHR TA VSMCs vs. WKY VSMCs. Given the evidence that integrins are the principal mediators of mechanical signaling in cell–ECM interactions (Chen, Tan & Tien, 2004; Ciobanasu, Faivre & Le Clainche, 2013; Coyer et al., 2012; Schwartz & DeSimone, 2008), the increased expression and activity of integrin β1 in SHR VSMCs provide a potential mechanism for higher cell–ECM interactions in the 3D reconstituted model and may represent a potential target for the amelioration of aortic stiffening.

Activated integrin β1 has been identified and reported in many studies through Western blotting by recognizing the activated epitope on integrin chain β1 (Lenter et al., 1993; She et al., 2010). Although it is generally accepted that integrin β1 can be activated from two directions: from the inside by the regulated binding of proteins to the cytoplasmic tails and from the outside by multivalent ligand binding (Arjonen, Alanko, Veltel & Ivaska, 2012; Legate & Fassler, 2009; Tadokoro et al., 2003), the underlying mechanisms are not fully understood in hypertension and ECM stiffening. Our current study puts forward a new concept that SRF/myocardin may act as a crucial mediator of the integrin β1 activation inside SHR TA VSMCs. Integrins can couple the cytoskeleton to ECM, thereby providing a mechanical connection between VSMCs and the extracellular environment. Our previous studies have shown that the expression of cytoskeletal proteins, such as smooth muscle α‐actin (α‐SMA), are significantly increased in VSMCs from stiffening aortas in both hypertensive and aging animals (Qiu et al., 2010; Zhou, Lee, Stoll, Ma, Wiener, et al. 2017). We also revealed that increased cytoskeletal proteins in SHR VSMCs were significantly repressed by the inhibitor of SRF/myocardin (Zhou, Lee, Stoll, Ma, Wiener, et al. 2017). In this study, we found that SRF/myocardin inhibition attenuated the activation of integrin β1 in SHR VSMCs, suggesting that SRF/myocardin, the cytoskeleton, and the activation of integrin β1 are all interlinked. Based upon the evidence from our previous studies and the results from the current study, it is reasonable to speculate that increased SRF/myocardin in SHR VSMCs governs the expression of cytoskeletal proteins, such as α‐SMA, which may alter their binding or interacting with the cytoplasmic tails of integrin β1, subsequently activating integrin β1 from the inside and inducing cell signaling between VSMCs and the extracellular environment in SHR aorta.

Our study also revealed a comprehensive regulation of LOX in VSMCs from SHR TA. First, we showed that SHR TA VSMCs increased the intracellular mRNA expression and the production of mature‐LOX along with regulatory LOX‐PP. These data indicate that SHR TA VSMCs exhibit a regulation of LOX at gene expression and proteolytic levels. Second, In contrast to previous studies, instead of a single mature‐LOX isoform as observed in most other tissues (Barker, Cox & Erler, 2012; Lopez et al., 2009; Lucero & Kagan, 2006; Payne, Hendrix & Kirschmann, 2007), we detected two forms of mature‐LOX in rat aortic VSMCs. This finding could imply that a unique mechanism may be involved in the proteolytic processing of LOX, by which different sites of pro‐LOX may be targeted and result in two different sizes of mature‐LOX. In addition, decreased LOX activity was found in the extracellular media of the SHR TA VSMCs vs. WKY. This may be due to the reduced extracellular release of LOX from SHR TA VSMCs. The decrease in extracellular LOX activity in SHR TA VSMC support previous studies showing LOX deficiency causes disorganized connective tissue formation *in vivo* (Lee et al., 2016; Maki et al., 2002; Staiculescu et al., 2017) and may also explain the disorganization of collagen observed in the reconstituted tissue with SHR TA VSMCs.

At present, conflicting data have been reported regarding the relationship between LOX and aortic stiffness in different models. For example, while it has been shown that the downregulation of LOX contributes to the aortic stiffening in an obesity mouse model (Chen et al., 2013), other studies have shown that inhibition of LOX attenuated the angiotensin II induced aortic stiffening (Eberson et al., 2015). In addition, as evidence showed that genetic deletion or functional deficiency of LOX causes aortic wall destruction and leads to aortic stiffening (Lee et al., 2016; Maki et al., 2002; Staiculescu et al., 2017), a smooth muscle cell‐specific overexpression of LOX in a mouse model has been shown to induces arterial stiffness (Martinez‐Revelles et al., 2017). Although the reasons for these inconsistencies are unclear, one potential explanation may arise from the distinct extracellular and intracellular effects of LOX. Although it has been widely accepted that mature active LOX functions in the extracellular cross‐linking of collagens and elastin, this active form of LOX has also been detected to be increased inside the VSMCs and is able to stimulate intracellular signaling pathways as detected in LOX transgenic mice (Martinez‐Revelles et al., 2017). In addition, it has been reported that LOX‐PP participates in the regulation of the focal adhesion kinase in cancer cells (Zhao et al., 2009), although its biological activity in VSMC remains to be identified. These observations suggest the existence of undefined roles of M‐LOX and LOX‐PP in the cellular function of VSMCs via intracellular effects, which may also involve the regulation of aortic stiffness. Furthermore, while the disturbances of LOX expression and function could induce disorganization of the ECM due to lower extracellular activity of LOX, overexpression of exogenous LOX in VSMCs may also induce abnormally high levels of extracellular LOX activity and cause excessive cross‐linking of collagen and lead to an increase in vascular stiffness. Our data from the current study showed that SHR VSMCs exhibit an increased mature active LOX and LOX–pp inside the VSMCs with a decrease in extracellular LOX activity, which supports the concept that LOX regulates hypertensive aortic stiffness through both intra‐ and extracellular mechanisms. Moreover, the regulation of other isoforms of LOX in aortic VSMCs is largely unknown and the potential complementary and/or competitive mechanisms among these isoforms may need to be investigated. Overall, the involvement of LOX in SHR VSMCs opens up new prospects in mechanistic studies of hypertensive aortic stiffening, which is likely mediated by both intracellular and extracellular disturbances.

Our study also revealed a potential link between the alterations of LOX and SRF/myocardin signaling. Inhibition of SRF/myocardin by CCG reversed increased proteolytic cleavage of LOX in SHR TA VSMCs with a slight effect on extracellular LOX activity. These data together indicate that the effect of CCG is predominantly on intracellular signal transduction rather than extracellular effects. Considering that SRF is a transcriptional factor, which is well known to be primarily involved in the regulation of gene expression rather than conducting proteolytic function itself, we further explored the mechanism by which SRF/myocardin regulates the maturation and activation of LOX in SHR VSMCs. Our results demonstrated that BMP1, a known protease of LOX, acts as a mediator between SRF/myocardin and LOX cleavage. In addition, inhibition of BMP1 specifically repressed the maturation and activation of LOX in SHR VSMCs vs. vehicle with no change in Pro‐LOX, which is consistent with the effect of CCG. These findings together indicate that SRF/myocardin may regulate the maturation of LOX via the regulation of BMP1. Most importantly, we showed that inhibition of SRF/myocardin by CCG dramatically reduced the stiffness of reconstituted tissue formed with SHR TA VSMCs.

It is also notable that, while CCG treatment significantly rectified the pathological alterations of integrin β1, BMP1, LOX, and stiffness in SHR TA VSMCs, the effects of CCG on WKY TA VSMCs are negligible vs. vehicle. This selective effect indicates that SRF/myocardin signaling may also be involved in the regulation of the extracellular effects in SHR VSMCs. This selective effect of CCG is consistent with our previous results showing the selective effects of CCG on aortic stiffness and blood pressure in SHR *in vivo* (Zhou, Lee, Stoll, Ma, Wiener, et al. 2017). Although the SRF/myocardin pathway has been reported as primarily regulating cytoskeletal proteins and involved in VSMC differentiation and stiffness (Zhou, Lee, Stoll, Ma, Wiener, et al. 2017), our data from this study demonstrate that SRF/myocardin signaling also participates in VSMC‐medicated extracellular dysregulation in hypertension, bringing new insight into the mechanism integrating intra‐ and extracellular function of VSMCs in aortic stiffening.

In summary, our results indicate a comprehensive regulatory mechanism induced by VSMCs in the stiffened aorta in hypertension. We put forward the concept that VSMCs are capable of driving aortic tissue stiffness by increasing the interaction between VSMCs and the ECM and altering ECM production and remodeling. These extracellular effects of aortic VSMCs are regulated by a signaling pathway, which involves upregulating SRF/myocardin, the cytoskeleton/integrin β1, and BMP1/LOX.

Although the association between aortic stiffness and hypertension is well established, whether aortic stiffening is a cause or a consequence of hypertension remains controversial. Our recent study demonstrated that aortic stiffness reduction preceded the reduction in blood pressure in SHR treated with CCG, suggesting the recovery of aortic elasticity in SHR is not likely an artifact of the reduction in blood pressure, and may even be a contributing factor. (Zhou, Lee, Stoll, Ma, Costa, et al. 2017; Zhou, Lee, Stoll, Ma, Wiener, et al. 2017). We believe that the mechanisms revealed in the current study may also contribute to aortic stiffness in aging, which allow us insight into the pathological development of hypertension in the elderly. In addition, the current study focused on the aortic VSMCs due to the observed aortic stiffening in SHR; however, our results should stimulate further investigation upon the VSMCs from smaller arteries as well. Furthermore, while we showed that VSMCs impact the ECM in stiffening SHR TAs, and reciprocally altered ECM may also impact the function of VSMCs in aortic stiffening, which represents an interesting topic for the future investigations.

## 4 EXPERIMENTAL PROCEDURES

### 4.1 Animal model

Adult (16–18 weeks old) male SHR and their normotensive controls, WKY rats (Charles River Laboratories, San Diego, CA, USA), were studied. All animal procedures were performed in accordance with the NIH guidance (Guide for the Care and Use of Laboratory Animals, revised 2011) and the protocols approved by the Institutional Animal Care and Use Committee of Loma Linda University.

### 4.2 Measurement of blood pressure (BP)

BP was measured in the conscious state by restraint tail cuff every 2 days for 2–3 weeks for the two experimental groups using a CODA system (Kent Scientific, Torrington, CT, USA). Direct BP measurement was performed under anesthesia with an inspired concentration of 2.5% isoflurane (JD Medical, Phoenix, AZ, USA) in the descending thoracic aorta accessed through the right common carotid artery by Millar catheter (Millar, Inc. Houston, TX, USA). The transducer was connected to a Powerlab system (AD Instruments, Castle Hill, Australia) to record systolic and diastolic aortic pressure (SAP and DAP) as described (Zhou, Lee, Stoll, Ma, Wiener, et al. 2017).

### 4.3 *In vivo* aortic stiffness measurements

Hemodynamic assessment was performed by a Doppler ultrasound echocardiography under anesthesia with 2.5% isoflurane (JD Medical, Phoenix, AZ, USA). All rats were subjected to ultrasound evaluation every week for 3 weeks as described previously (Zhou, Lee, Stoll, Ma, Wiener, et al. 2017), and the average value was calculated from the three timed measurements. Aortic stiffness was calculated using the following equations: ß = Ln[(SBP/DBP)/(ΔD/D)]), and E_Y _= D/h/DC), where SBP: systolic blood pressure; DBP: diastolic blood pressure; D: diastolic diameter of the artery; ΔD: systolic minus diastolic diameter change; h: the thickness of aortic wall; DC: (distensibility) =ΔA×PP/A; A: the minimal cross‐sectional area of the aorta, ΔA: the maximal minus minimal cross‐sectional area of the aorta; and PP: pulse pressure (Zhou, Lee, Stoll, Ma, Wiener, et al. 2017).

### 4.4 *Ex vivo* aortic stiffness measurements

Rat descending TAs were harvested and immersed in ice‐cold Krebs buffer solution containing (in mm) 122 NaCl, 25.6 NaHCO3, 5.17 KCl, 2.49 MgSO4, 1.60 CaCl2, 2.56 dextrose, 0.027 EGTA, and 0.114 ascorbic acid. Aortas were then cleaned of extraneous connective tissue and denuded of the endothelial layer by rubbing the intimal surface with a wire. The vessel segments (2–3 mm in length) were mounted onto wires connected to an isometric force transducer (model 52‐9545, Harvard Apparatus, South Natick, MA, USA) and suspended in a sodium‐replete Krebs buffer solution (pH 7.4) at 37°C and bubbled with 95% O_2_ and 5% CO_2_. The vessel segments were then subjected to uniaxial tensile stretching in a stepwise fashion from 20% to 140% of their original resting length, and the resting stress was recorded at each step. The *ex vivo* aortic stiffness (E) was calculated using the formula E=F*L/(A*ΔL) based on the circumferential stress of the vessel segment, where F is the difference in steady‐state force at each stretch level, A is the cross‐sectional area of the segment, L is the original length of the tissue, and ΔL is the stretched length of the tissue. A stress–strain plot was generated from these experiments, and the slope of the line was used to compute the tangential elastic modulus.

### 4.5 VSMC isolation, culture, and treatments

Primary VSMCs were isolated from the TA of SHR and WKY rats by natural migration as done previously (Zhou, Lee, Stoll, Ma, Wiener, et al. 2017). These isolated cells were serially cultured to 3 or 4 passages and were treated with CCG‐100602 (25 μm) (Cayman Chemical, Ann Arbor, MI, USA) or UK 383367 (5 um/L) (TOCRIS, MN, USA, Cat.4188) or vehicle control (DMSO, Sigma‐Aldrich, St. Louis, MO, USA) for 24 hr and then were collected for RNA and protein extraction or immunostaining (Zhou, Lee, Stoll, Ma, Wiener, et al. 2017).

### 4.6 *In vitro* VSMC stiffness measured by 3D reconstituted tissue model

Vascular smooth muscle cells were encapsulated in collagen gels (1 mg/ml) at a seeding density of (750,000 cells/ml) and allowed to congeal around a cylindrical mandrel and cultured for 48 hr. The resulting artificial segments were then removed from the mandrel and mounted onto wires connected to an isometric force transducer (model 52‐9545, Harvard Apparatus, South Natick, MA) and suspended in an in a sodium‐replete Krebs buffer solution (pH 7.4) at 37°C and bubbled with 95% O_2_ and 5% CO_2_. The artificial vessel segments were then subjected to uniaxial tensile stretching in a stepwise fashion from 3% to 21% of their original resting length and the resting stress was recorded at each step. The construct's stiffness (E) was calculated for each stretch as described above in the native aortic ring.

### 4.7 Histological and image analysis

Z‐stack images ~50 um thick of artificial vessel segments were acquired using two‐photon confocal microscopy on a Zeiss LSM 710 NLO microscope to construct a 3D model of the artificial ring. The artificial vessel ring collagen was detected using second harmonic generation (SHG) microscopy, and VSMCs were stained for α SMA (A2547 Sigma‐Aldrich, St. Louis, MO, USA) and then imaged (Jena Germany) as described (Grossman et al., 2016). Volocity image analysis software (Perkin Elmer, Waltham, MA, USA) was then used to segment the VSMCs in 3D based upon a‐SMA staining and calculate the respective parameters of VSMCs automatically with the captured 3D cell images (Porter, Holt, Soong, Shanahan & Warren, 2016). Artificial vessel image acquisition was carried out upon longitudinal sections of tissue and therefore perpendicular to the stretch axis. Cell diameter is defined as the diameter of a cylinder equal to the cell volume along the cell's longest axis. Cell roundedness (or Shape factor) is calculated as the ratio of the surface area of a sphere with the same volume as the cell over the surface area of the cell. Imaging analysis for collagen directionality was done by Fiji package (Schindelin et al., 2012). Fourier component analysis for directionality was performed on data using the Fiji plug‐in “Directionality” and following manufacturer's instructions (Grossman et al., 2016).

### 4.8 RNA extraction and real‐time PCR

RNA was extracted from isolated VSMCs using the Quick‐RNA MiniPrep kit (Genesee Scientific, San Diego, CA, USA) according to the manufacturer's instruction. RNA concentration was determined through photometric measurement on the Nanodrop 2000 (Peqlab, Erlangen, Germany). Quantitative real‐time PCR was performed on a CFX96 Touch™ Real‐Time PCR Detection System using iTaq™ Universal SYBR^®^ Green Supermix (Bio‐Rad, Hercules, CA, USA) according to the manufacturer's manual. All real‐time PCRs were performed in triplicate (Zhou, Lee, Stoll, Ma, Wiener, et al. 2017).

### 4.9 Protein extraction and Western blot

Total protein was extracted from VSMCs using cell extraction buffer (Cat No. FNN0011, life technology, Carlsbad, CA, USA) and then measured by Western blotting and detected using a LI‐COR Odyssey^®^ Infrared Imaging System (Lincoln, NE, USA) (Zhou, Lee, Stoll, Ma, Wiener, et al. 2017).

### 4.10 LOX activity measurement

Lysyl oxidase activity was measured in cultured medium using LOX activity Assay Kit (Abcam, Cambridge, MA, USA) according to the instructions of the kit. The assay measured LOX‐dependent H_2_O_2_ release from a proprietary substrate, followed by fluorescent detection of H_2_O_2_. Increases in fluorescence were detected at Ex/Em = 540/590 nm in a fluorescence microplate reader (Molecular Devices, Spectramax i3x, Dublin, CA, USA).

### 4.11 Statistical analysis

Results are presented as the mean ± *SEM* for the number of samples indicated in the figure legends. One‐way ANOVA or two‐way ANOVA was used to test for significance between groups. Student–Newman–Keuls post hoc correction was applied for multiple pairwise comparisons. A value of *p < *.05 was considered statistically significant.

## CONFLICT OF INTEREST

None.

## AUTHOR CONTRIBUTIONS

H.Q. conceived and designed the study. H.Q., T.H., B.M., and W.P. designed and performed *ex vivo* and *in vitro* experiments, analyzed the data, and wrote the manuscript; N.Z. and S.S designed, performed, and analyzed the *in vivo* experiments. All authors discussed and analyzed the results and reviewed and approved the final version of the manuscript.
